# Enzyme Immobilization on Nanomaterials and Their Applications

**DOI:** 10.3390/ma18174106

**Published:** 2025-09-01

**Authors:** Rahul Vikram Singh, Bakul Singh, Anurag Kumar, Krishika Sambyal, Karthikeyan Kugalur Karuppanan, Jung-Kul Lee

**Affiliations:** Department of Chemical Engineering, Konkuk University, Hwayang-dong, Gwangjin-gu, Seoul 143-701, Republic of Korea; rahul.negi121@gmail.com (R.V.S.); bakulsingh.singh8@gmail.com (B.S.); anuragbioinfo.2017@gmail.com (A.K.); krishika.sambyal@gmail.com (K.S.); karthikk1529@gmail.com (K.K.K.)

**Keywords:** immobilization, biotransformation, nanomaterial, magnetic nanoparticle, reusability

## Abstract

Immobilizing enzymes onto nanomaterials is a promising approach for increasing the efficiency of industrial biotransformation processes. Nanomaterials have large surface areas and unique physicochemical characteristics, they increase enzyme stability and catalytic efficiency, and they can be reused multiple times, making them favorable over free enzymes. Various nanomaterials, including carbon-based materials, metal and metal oxide nanoparticles, and polymeric nanoparticles, have been explored for immobilizing enzymes. Immobilized enzymes are more reusable than free enzymes, which are suitable for industrial applications such as in pharmaceuticals, as drug intermediates, and for synthesizing fine chemicals. Using immobilized enzymes multiple times enables numerous catalytic reactions, substantially increasing product yield and minimizing enzyme consumption, thus optimizing process efficiency and cost-effectiveness of manufacturing processes. This review explores recent developments in nanomaterials for immobilizing enzymes and biotransformation.

## 1. Introduction

Immobilizing enzymes on nanomaterials is a promising approach for increasing the efficiency of biotransformation processes in various industries. This technique combines the catalytic properties of enzymes with the unique properties of nanomaterials, resulting in increased enzyme stability, activity, and reusability, and provides large surface area for efficient immobilization, offering advantages over traditional enzymatic applications [1,2]. Enzymes are biological (natural and engineered) catalysts that play crucial roles in numerous industrial processes, including biofuel production, food processing, and pharmaceutical manufacturing [1]. However, the limited stability, recovery and reuse difficulties, and susceptibility to environmental factors hinder the use of free enzymes in industrial applications [2]. Enzymes have been immobilized on solid supports, particularly nanomaterials, to address these limitations and to improve the overall efficiency of enzymatic processes [3].

**Figure 1:**
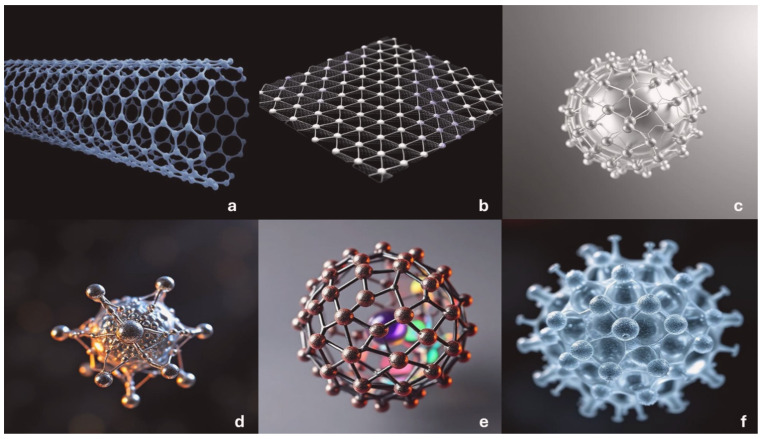
AI-generated 3D structures of different nanoparticles: (**a**) carbon nanotube (CNT); (**b**) graphene; (**c**) silver nanoparticle; (**d**) magnetic nanoparticle (MNP); (**e**) metal–organic framework (MOF); and (**f**) polymeric nanoparticle. Figure 1 was generated through deep artificial intelligence by authors.

Nanomaterials have received considerable attention as support for immobilizing enzymes owing to their large surface-area-to-volume ratio, high biocompatibility, and physicochemical characteristics [4]. Using nanomaterials as enzyme carriers can considerably increase enzyme stability, catalytic activity, and reusability compared with enzymes used without these carriers, enabling the development of more cost-effective and sustainable industrial processes [1,2]. Various types of nanomaterials have been tested for their potential in enzyme immobilization, including carbon-based materials such as carbon nanotubes (CNTs) and graphene, silver nanoparticles, magnetic nanoparticles (MNPs), metal–organic frameworks (MOFs), and polymeric nanoparticles, as illustrated in Figure 1 [2,4]. Each type of nanomaterial offers distinct advantages and disadvantages, as described in Table 1, and each can be tailored to suit specific enzyme immobilization requirements [3].

**Figure 2:**
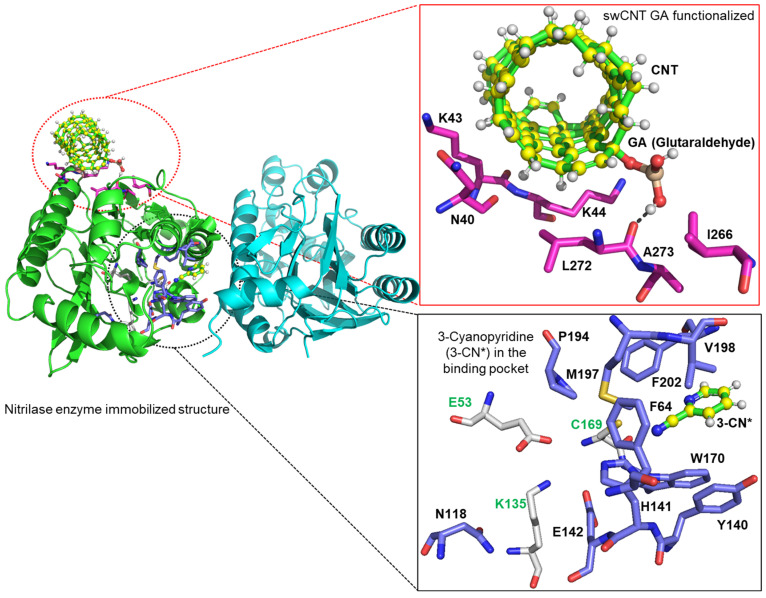
Interaction between nitrilase, substrate, and immobilized matrix. (Nitrilase (pdb: 3wuy) is immobilized on a glutaraldehyde (GA)-functionalized single-walled carbon nanotube (swCNT). GA interacts with enzyme surface residues, including a hydrogen bond with the backbone carbonyl oxygen of A273 and hydrophobic contacts with residues K44, K43, and N40 (inset, top). The substrate, 3 cyanopyridine, binds productively in the enzyme pocket (inset, bottom), stabilized by π–π interactions with W170, F64, and F202, which orient the cyano group near the catalytic residue C169 for nucleophilic attack. The active site residues E53 and K135 further stabilize the transition state. This model highlights the molecular interactions enabling catalysis upon immobilization).

Enzymes and substrates must interact to produce catalytic activity. The induced-fit hypothesis states that the enzyme and substrate undergo conformational changes upon binding, ensuring catalysis. Immobilizing enzymes can affect these interactions. Although the activity of immobilized enzymes may be lower owing to mass transfer barriers, immobilized enzymes are often more stable across broader pH and temperature ranges. A specific distance is required between the immobilized enzymes and the support surface to ensure structural stability [12]. The immobilization method, such as covalent attachment or adsorption, influences the enzyme–substrate interactions [13]. The hydrophobic nature of the carriers stabilizes enzyme, and multipoint attachment increases thermal stability, which ensures enzyme–substrate interactions as well as increasing enzyme stability and reusability [14,15,16,17]. Understanding the interactions between the enzyme, substrate, and immobilized matrix is crucial, as matrix compatibility influences how the matrix binds to the enzyme and determines the type of bonding that provides high stability during enzyme–substrate reactions. For example, computational studies have examined nitrilase (pdb:3wuy) immobilized on single-walled carbon nanotubes (swCNTs) functionalized with glutaraldehyde (GA) in this regard, as shown in Figure 2. In this setup, the immobilization matrix (yellow-green) offers structural support while preserving enzyme activity. The binding pocket of the enzyme accommodates the 3-cyanopyridine substrate, which interacts with key amino acid residues, such as P194, M197, V198, F202, C169, W170, H141, Y140, E142, K135, N118, and E53. Notably, aromatic residues, such as W170, F64, and F202, form pi–pi interactions with the benzene ring of the substrate, positioning the cyanocarbon near the active-site residue C169, which then initiates catalysis through nucleophilic attack. Additional residues, including E53 and K135, help stabilize the transition state, and the optimal positioning of the substrate within the active site highlights the complementarity and multiple noncovalent interactions that facilitate efficient catalytic conversion of the nitrile compound [18,19]. Magnetic nanoparticles (MNPs) are a particular subject of interest for enzyme immobilization because of their superparamagnetic properties. Magnetite (Fe_3_O_4_) is the most widely used MNP for immobilizing enzymes owing to its cost-effectiveness, biocompatibility, low toxicity, and high magnetic susceptibility [20]. The use of MNPs facilitates the separation and recovery of immobilized enzymes from reaction mixtures using an external magnetic field, simplifying downstream processing [16].

**Figure 3:**
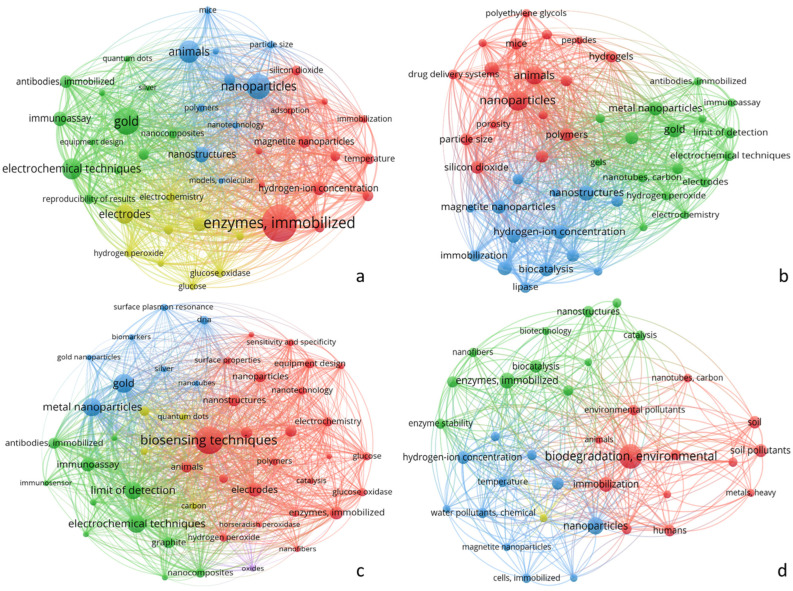
Evolution of nanomaterial application trends across four sectors (2001–2025). Network maps of keywords from PubMed reveal the growth and specialization of nanomaterial research in (**a**) biotransformation (highlighting enzymes, immobilization, and biological interfaces), (**b**) pharmaceutical sector (focusing on drug delivery and tumor targeting), (**c**) biosensor development (emphasizing biosensing, electrochemical techniques, and detection sensitivity), and (**d**) bioremediation (centered on environmental degradation and pollutant removal). The node size reflects the keyword importance, and the colors indicate the related groups, illustrating the expanding and diverse roles of nanomaterials over 24 years.

Several immobilization techniques have been developed to attach enzymes to nanomaterials, including covalent, noncovalent, and encapsulation methods [21]. Each immobilization method has advantages and limitations (Table 2), and the choice of method depends on the specific enzyme, nanomaterial, and intended application [2]. For example, covalent binding provides strong attachment but alters the structure of the enzyme, whereas noncovalent methods preserve the enzyme structure, but enzyme leaching is problem [2,20]. Immobilizing enzymes on nanomaterials has improved enzyme performance in various biotransformation processes. Immobilized enzymes are often more stable under harsh conditions, such as at extreme pH or temperature, compared with their free counterparts [1,3]. This increased stability allows for the use of enzymes in a broader range of industrial applications and extends their operational lifetimes [11]. Moreover, immobilizing enzymes on nanomaterials increases catalytic activity and efficiency [22]. The large surface area of nanomaterials allows for the loading of large amounts of enzyme, and the properties of nanomaterials create a favorable microenvironment for enzyme function [4,23]. In some cases, the catalytic activity of immobilized enzymes is several-fold higher than that of free enzymes [3]. The field of immobilizing enzymes on nanomaterials continues to evolve, with advancements focusing on developing multifunctional nanomaterials and the co-immobilization of multiple enzymes [6,24] to create more efficient and versatile biocatalytic systems for complex biotransformation processes [23]. The number of applications of enzyme–nanomaterial conjugates is annually expanding across a wide range of industries. Figure 3 depicts the keyword networks and the focus of nanomaterial applications in different sectors since 2001. In Figure 3, the applications of nanoparticles are divided into four sections: (a) biotransformation, (b) pharmaceutical sector, (c) biosensor development, and (d) bioremediation. Therefore, on the PubMed platform, keywords, such as the “application of nanoparticle,” in all four sectors were searched, and all available data (research, reviews, and other articles) were analyzed using VOSviewer (version_1.6.20_exe) to generate the network. Initial application searches focused on biotransformation and pharmaceuticals because article searches revealed that most published studies on enzyme immobilization focused on the synthesis of pharmaceutically relevant drug intermediates, precursors, and fine chemicals. Subsequently, nanoparticles have been utilized in drug delivery systems in the pharmaceutical sector. The development of nanomaterial-based biosensors has gained traction, particularly in bioremediation, highlighting their expanding role in environmental solutions. This evolution reflects the broadening scope and advancing sophistication of nanomaterial research across various industries.

Nanomaterials have been used in biofuel production, and immobilized enzymes have shown promise in increasing the efficiency of biomass conversion and biocatalytic processes [26]. Immobilized enzymes are used in various food industry applications, such as the production of functional foods and modification of food components [28]. However, several challenges remain despite the progress in this field: optimizing immobilization protocols to maintain enzyme activity, increasing the long-term stability of immobilized enzymes, and addressing potential environmental and safety concerns associated with nanomaterials [3,8].

Classical supports generally provide strong stability and reliable interactions with enzymes but often suffer from limited surface area and less tunable properties. In contrast, nanomaterials offer significantly higher surface areas, customizable sizes and shapes, and enhanced surface chemistry. These features improve enzyme loading, activity, and stability by enabling stronger interactions and better substrate accessibility. The advanced properties of nanomaterials thus lead to superior efficiency over classical supports in catalytic and biotransformation processes, aligning with recent findings in nano-biocatalysis research [6,8,10].

## 2. Nanomaterials for Immobilizing Enzymes

**Figure 4:**
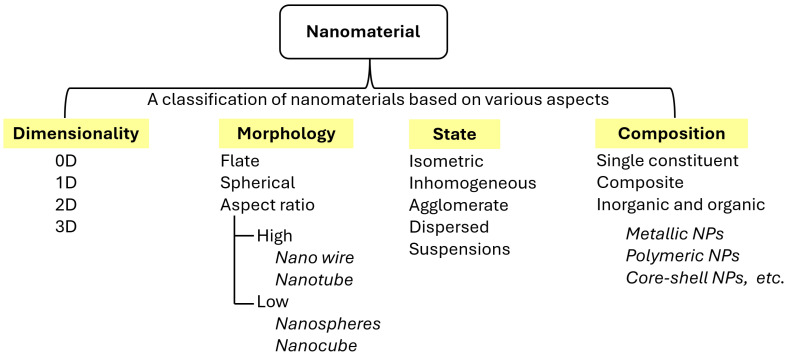
Classification of nanomaterials according to different features and their categorization.

Various types of nanomaterials with properties appropriate for immobilizing enzymes have been synthesized. Nanomaterials are classified according to their dimensionality, morphology, and chemical composition (Figure 4) [29]. These classes include zero-dimensional (0D) structures, such as nanoparticles and quantum dots; one-dimensional (1D) structures, such as nanotubes and nanowires; two-dimensional (2D) structures, such as graphene; and three-dimensional (3D) nanostructures [6,8,9,29]. The chemical composition of these nanomaterials ranges from carbon-based (fullerenes and CNTs) to metal-based materials (gold and silver nanoparticles), metal oxides (titanium dioxide and iron oxide), and organic materials such as dendrimers [15,25,30]. The classification can also be based on specific properties such as optical, electrical, and magnetic characteristics. However, defining nanomaterials remains challenging because of their dynamic nature and the limitations of measurement methods, leading to ongoing debates regarding their classification criteria [13,31,32].

### 2.1. Carbon-Based Nanomaterials

Carbon-based nanomaterials, particularly CNTs and graphene, have emerged as appropriate supports for immobilizing enzymes because of their properties. CNTs, both single-walled (swCNTs) and multiwalled (mwCNTs), have large surface areas and are chemically inert as well as biocompatible, so are suitable for immobilizing enzymes [33,34,35,36]. Functionalization of carbon nanotubes (CNTs) with different groups, such as amino, carboxyl, or silane groups, enhances their surface properties for enzyme immobilization. These modifications enable covalent binding or crosslinking; improve enzyme stability, activity, and reusability; and reduce leaching, making CNTs effective supports for biocatalysts, as described in Table 3 along with the nanomaterial, functional groups, enzymes, enzyme sources, and their reusability cycles in the reaction. CNTs have been commercialized because of their biocompatibility, biodegradability, and strong film-forming ability; therefore, CNTs have received considerable research attention [37,38]. CNT applications are not limited to enzyme immobilization but also include other fields, such as biosensor development. Integrating molecularly imprinted polymers (MIPs) with CNTs has expanded their potential for use in sensor development. CNT-based sensors show promise in medical diagnostics [39,40,41], environmental monitoring [42,43], and industrial processing [44,45]. CNT-based biosensors offer innovative solutions for immobilizing enzymes and detecting biomolecules. Carbon nanotube (CNT)-based nanomaterials have significant potential for applications in biotransformation by leveraging enzymatic and microbial processes to modify their structures and reduce toxicity. For instance, fungi such as *Penicillium chrysogenum* and Pleurotus ostreatus utilize ligninolytic enzymes (e.g., laccases and peroxidases) to oxidize and shorten multi-walled CNTs (MWCNTs), alter surface functional groups (C=O and O–H), and enhance biocompatibility. Similarly, microalgae, such as *Desmodesmus subspicatus* can remove up to 55% of absorbed CNTs from aquatic environments, indicating their potential for applications in environmental remediation. Surface engineering techniques such as PEGylation further optimize the stability and biodegradability of CNTs, which are crucial for biomedical and ecological applications. These biotransformation strategies enable safer CNT integration in drug delivery, pollution mitigation, and regenerative medicine [46,47]. Superparamagnetic mwCNTs with four-armed polyethylene glycol were used to covalently coimmobilize *Rhizopus oryzae* and *Candida rugosa* lipases for producing biodiesel from waste oils. Biodiesel is a renewable fuel produced by the enzymatic transesterification of waste oils using lipases such as *Rhizopus oryzae* and *Candida rugosa*. In this process, waste oil serves as a substrate for immobilized enzymes, which are carried on modified CNTs to enhance their efficiency and stability. When applied to biodiesel production from waste cooking oil under ultrasound assistance, this system achieved a 97.64% conversion rate in 120 min, owing to the synergistic action of both the lipases and ultrasound. Notably, the system maintained a yield of over 78.55% after 10 cycles, demonstrating excellent operational stability and significant potential for industrial biodiesel production and other biocatalytic applications [48]. Other studies have proved that enzymes immobilized on CNTs retain high catalytic activity and multiple cycles of enzyme reusability (Table 3). The curvature of the nanotubes affects the enzyme immobilization yield and behavior, with enzymes covalently immobilized on amine-functionalized CNTs having higher catalytic activity and operational stability than physically adsorbed enzymes.

### 2.2. Graphene and Derivatives

Graphene and its derivatives have diverse applications in biomedicine, energy science, and material sciences. Because of their unique properties, they are used in biosensing, bioimaging, drug delivery, and tissue engineering. Graphene-based materials have been used in energy applications to enhance the performance of solar cells, batteries, and supercapacitors. They are suitable for nanoelectronics, sensors, and composite materials owing to their mechanical and electrical properties [70,71,72]. Functionalization of graphene and its derivatives with various groups (e.g., carboxyl, hydroxyl, and epoxy) enhances its surface chemistry, enabling effective enzyme immobilization. These modifications improve enzyme stability, activity, and reusability by promoting strong physical or covalent attachment, making graphene-based materials excellent carriers for biocatalysts, and such materials are presented in Table 4, including enzymes and their sources, functionalization groups, recyclability, and retained activity. Chloroperoxidase (CPO) and glucose oxidase (GOx) were co-immobilized on magnetic graphene oxide (MGO) to increase its catalytic activity. The activity of MGO-GOx-CPO was higher (96.6%) than that of separately immobilized enzymes (86.2%) in orange G decolorization. The co-immobilized system was more thermally stable and 38.5% of the activity was maintained after six reuse cycles. This approach demonstrates potential for environmental applications like wastewater treatment, combining efficient catalysis with easy magnetic recovery [73]. Metal–organic frameworks (MOFs) and graphene oxide (GO) have been combined with single-stranded DNA as a linker to co-immobilize glucose oxidase (GOx) and horseradish peroxidase (HRP). This approach had an enzyme stability of <70% of the initial activity and achieved activity through π–π stacking as well as coordination bonding. The immobilized GOX–HRP system showed enhanced in cascade reaction kinetics, substrate selectivity, and a wide linear range (50–750 μM) for detecting glucose. The reusability of PCN-222 and GO@DNA@GOx, HRP (PGE), and PCN-222 and GO@enzymes was investigated. PGE retained more than 75% catalytic activity after 10 cycles, whereas PCN-222 and GO@enzymes retained less than 30% of their initial activities [73]. A capillary-electrophoresis-based dual-enzyme microreactor (THR-FXa IMER) was developed using a polydopamine/GO coating to co-immobilize thrombin and factor Xa. The system accurately measured enzyme kinetics and determined inhibition constants and maintained 98% activity after 30 cycles. The system successfully screened dual-target inhibitors from 30 compounds, and the results of molecular docking validated these interactions, leading to the design of a new anticoagulant compound. THR-FXa IMER is a reliable method for screening coagulation enzyme inhibitors and aided in the development of anticoagulant drugs [74]. Graphene-based systems, such as MGO-GOx-CPO and MOF-GO hybrids, are catalytically active, stable, and reusable when immobilizing enzymes, demonstrating potential for remediating the environment and detecting glucose. Additionally, the dual-enzyme microreactor (THR–FXa IMER) highlights the role of graphene in drug discovery, as the use of graphene has enabled the efficient screening of anticoagulant compounds. These findings underscore the importance of graphene-based materials for creating sustainable high-performance platforms for diverse scientific and industrial applications. Graphene-based materials exhibit versatility in immobilizing enzymes, having increased catalytic performance, stability, and reusability compared with prior materials. The success of enzymes co-immobilized on graphene derivatives (Table 4) highlights their synergistic effects and ability to increase the overall efficiency of these systems. The literature underscores the value of graphene in developing sustainable high-performance platforms for various scientific and industrial applications [75,76,77,78,79,80].

### 2.3. Metal and Metal–Oxide Nanoparticles

Metals such as Ag, Fe, Au, Cu, Pt, Zn, Mg, and Mn as well as metal–oxide nanoparticles such as TiO_2_, ZnO, Fe_3_O_4_, CuO, Al_2_O_3_, and MgO are suitable supports for immobilizing enzymes owing to their properties [109,110]. These nanoparticles have large surface-area-to-volume ratios, show superparamagnetic behavior (Fe_3_O_4_), and are easy to functionalize, suitable for increasing enzyme stability and catalytic activity. Fe_3_O_4_ nanoparticles are particularly popular owing to their biocompatibility, low toxicity, and low cost. Fe_3_O_4_ is easily separated and recovered using external magnetic fields when used for immobilizing enzymes, increasing the reusability of the enzymes used in industrial processes and protecting enzymes from harsh environmental conditions [111]. Surface modifications, such as coatings with various functional groups including thiols, amines, and carboxyls, enhance their stability, solubility, and biocompatibility, enabling efficient enzyme immobilization. These modifications facilitate strong enzyme attachment, improve catalytic performance, and expand applications in biosensing and biocatalysis, which are described in Table 5, along with enzymes, their sources, functionalization groups, and recyclability with retained activity. The enzyme immobilization on these nanoparticles are more thermally stable, tolerant to pH, and stable during storage than free enzymes, with some retaining up to 80% activity after extended storage periods (Table 5) [112]. The use of metal and metal–oxide nanoparticles, such as Fe_3_O_4_, TiO_2_, and CuO, has advanced enzyme immobilization by increasing enzyme stability, reusability, and catalytic activity. Their properties, including large surface area and functionalization potential, enable their applications in biosensing, environmental remediation, and biocatalysis. Advanced platforms that integrate nanoparticles with enzymes are highly sensitive, selective, and practical for industrial and biomedical applications [17,113,114,115,116,117,118,119,120].

### 2.4. MNPs

MNPs are composed of elements such as iron, cobalt, nickel, gadolinium, manganese, and chromium, and exhibit unique magnetic properties when exposed to external magnetic fields. These nanoparticles offer substantial advantages over other nanomaterials primarily because of their ease of separation from reaction mixtures by applying magnetic fields. This feature enables the efficient recovery and reuse of MNPs in various applications [130]. MNPs also have a large surface area-to-volume ratio, which enhances their catalytic activity and suitability for immobilizing enzymes. Magnetic nanoparticles (MNPs) are functionalized with various groups, such as amines, carboxyls, aldehydes, or peptide-based linkers, to enhance enzyme immobilization. These modifications improve the enzyme binding, stability, and reusability, enabling efficient catalytic applications and easy magnetic recovery in industrial processes. A few significant studies are reported in Table 6, along with various enzymes and their sources, functionalization groups, recyclability, and retained activity. Their superparamagnetic behavior, biocompatibility (especially with iron-oxide-based MNPs), and versatile surface functionalization have further contributed to their widespread use in fields such as biomedicine, industrial catalysis, environmental remediation, wastewater treatment, and pollutant removal [131,132,133]. More recent advancements have focused on developing MNPs tailored for specific applications, optimizing enzyme immobilization protocols, and creating enzyme-based bioreactors for industrial use [20].

The versatility of MNPs continues to drive innovation in fields ranging from biosensing to environmental remediation. Patil et al., (2022) [148] studied the co-immobilization of glucoamylase and α-amylase enzymes onto magnetic nanoparticles (MNPs) using glutaraldehyde as a cross-linking agent for the pre-treatment of *Curcuma longa* powder. The process was optimized for maximum enzyme activity, achieving the best results at a 1:4 MNPs-to-enzyme ratio, 60 mM glutaraldehyde concentration, and 120 min of cross-linking. The resulting enzymes@AMNPs were spherical with an average size of 100 nm and exhibited strong superparamagnetic properties (36.1 emu/g). When combined with low-power ultrasound, the biocatalyst significantly enhanced curcuminoid extraction yields by 1.3–1.5 times compared to individual methods. Subsequent crystallization led to a 54% (w/w) isolation of curcuminoids with 91% purity. The biocatalyst also demonstrated excellent reusability, maintaining about 50% activity after 10 cycles and over 95% activity after 30 days of storage [148]. Cascade reactions catalyzed using multienzyme systems offer considerable advantages over single-enzyme systems in industrial applications. Hydroxylase monooxygenase components (HpaB and HpaC) were co-immobilized on Ni-NTA-functionalized magnetic silica nanoparticles (Ni-NTA/H_2_N-SiO_2_@Fe_3_O_4_) to increase enzyme stability and activity. The co-immobilized system exhibited 2.6-fold higher activity than that with free enzymes, retained 76.6% activity after 12 days of storage, and maintained over 60% activity after seven cycles. This approach shows potential for industrial biocatalytic applications [149]. *Thermomyces lanuginous* lipase (TLL), *Candida antarctica* lipase B (CALB), and *Rhizomucor miehei* lipase (RML) were co-immobilized on amine-functionalized silica-coated magnetic nanoparticles via multicomponent reactions. The stability and specific activity of the co-immobilized enzymes were higher than those of the free enzymes. These co-immobilized enzymes were used for catalyzing biodiesel production from waste cooking oil, with Fe_3_O_4_@SiO_2_-NH_2_-RML-CALB and Fe_3_O_4_@SiO_2_-NH_2_-TLL-CALB achieving maximum FAME yields of 99% and 80%, respectively, under the optimized conditions determined using the response surface methodology and a central composite rotatable design [150].

MOFs are suitable carriers for immobilizing enzymes, although challenges such as enzyme conformational changes and substrate mass transfer resistance remain. Researchers developed a magnetic, hierarchical porous MOF (mH-UiO-66(Zr)) to co-immobilize enzymes horseradish peroxidase (HRP) and glucose oxidase (GOx. This design minimized enzyme leakage, reduced mass transfer resistance, and simplified handling via magnetism. The resulting bienzyme bioreactor exhibited exceptional stability (95.1% activity at 70 °C, >90% after 15 cycles), enhanced substrate affinity, and effectively degraded/transform pollutant 2,4-DCP, showcasing environmental remediation potential [151]. A novel magnetic four-enzyme nanobiocatalyst was developed through c-immobilizing cellulase (CelDZ1), β-glucosidase, GOx, and HRP onto amino-functionalized MNPs. The nanobiocatalyst exhibited high thermal stability, retaining up to 50% activity after five cycles and remaining active after 24 days at 5 °C. This nanobiocatalyst was successfully applied in a four-step cellulose hydrolysis cascade reaction, demonstrating its potential for industrial biocatalysis [152,153]. Qiu et al. (2021) [153] developed a novel biocatalyst by co-immobilizing laccase and 2,2-binamine-di-3-ethylbenzothiazolin-6-sulfonic acid on chitosan MNPs modified with an amino-functionalized ionic liquid (MACS-NIL-Cu-lac). The biocatalyst improved 1.7-fold activity as well as high stability and pollutant removal efficiency, achieving 100% 2,4-dichlorophenol and bisphenol A removal efficiency. The biocatalyst retained 93.2% efficiency after six cycles, demonstrating potential for practical water treatment applications [153]. MNPs effectively co-immobilize multiple enzymes and improved the catalytic activity, stability, and reusability (Table 6). MNP applications range from industrial biocatalysis to environmental remediation, showing potential for creating efficient and sustainable enzymatic systems in various fields [154,155,156,157,158].

### 2.5. Polymeric Nanoparticles

Polymeric nanoparticles are versatile platforms for immobilizing enzymes (Table 7), offering numerous advantages, such as high surface area, tunable properties, and biocompatibility. Polymeric nanoparticles can be synthesized from various natural and synthetic polymers, including polysaccharides, polystyrene, and polyurethane, providing a stable microenvironment for enzymes [159]. Functionalization of polymeric nanoparticles with various functional groups, such as amines, carboxyls, thiols, or biomolecular ligands, enables efficient enzyme immobilization. Techniques such as covalent binding, click chemistry, and surface adsorption allow precise attachment, enhancing the versatility and stability of NPs for biocatalysis, targeted delivery, and biosensing applications. Multiple functional groups can be incorporated in a single step for multifunctional use; for example, hyaluronic acid, chitin, chitosan, chitosan-cobalt oxide beads, and chitosan-coated superparamagnetic nanoparticles are described in Table 7, along with their enzymes, sources, functionalization groups, and recyclability with retained activity [160]. Suthiwangcharoen et al. (2014) developed novel core–shell nanoparticles using poly(4-vinylpyridine) to co-immobilize GOx and HRP, resulting in a 20% increase in enzyme activity compared with that of free enzymes [161]. Lipase was immobilized on polyurethane nanobiocatalysts via immobilizing CalB lipase on polyurethane (PU) nanoparticles functionalized with poly(ethylene glycol) (PU-PEG) synthesized via miniemulsion polymerization. The resulting p-NPB hydrolysis, enantioselective Mandelic acid hydrolysis, and were-3 ethyl ester production were analyzed. The kinetic parameters of PU-PEG6000-CalB exhibited the highest, whereas the activity of PU-PEG400-CalB was the highest, producing 43.72 and 16.83 mM.U^−1^ ethyl esters using EPA and DHA, respectively.

Trehalose has high thermal stability, and trehalose nanobiocatalysts show potential for use in sustainably synthesizing food and pharmaceuticals [162]. Polymeric nanoparticles show considerable potential in various applications for immobilizing enzymes, including biocatalysis, biosensing, and bioremediation. These systems exhibit high enzyme stability, reusability, and prolonged activity under harsh conditions. For example, Lee et al. (2008) [163] immobilized lipases on polyaniline nanofibers with iron oxide. The immobilized lipases were highly stable, easy to recover, and reusable, retaining over 80% activity after 32 days at room temperature. The system was applied for enantioselective esterification, yielding 18% prophilic ester of ibuprofen from racemic ibuprofen after 96 h, demonstrating an ability to efficiently and selectively perform biocatalysis [163]. However, challenges with mass transfer and scalability require ongoing research in the field of immobilizing enzyme using polymeric nanoparticles [164,165,166,167,168].

**Table 7:** Polymeric nanoparticles used for immobilizing enzymes.

Nanomaterial	Skeleton Matrix	Functionalization Groups	Enzyme	Enzyme Source	Reusability (Retained Activity)	Reference
**Polymeric nanoparticles**	Hyaluronic acid, chitin, and chitosan	Glutaraldehyde	Catalase	Sunflower seeds	25 cycles (73.80%) (79.55%)	[169]
	CS-ALG-Fe_3_O_4_ MNPs	Ionic gelation method	Laccase	*Trametes versicolor*	10 cycles (81%)	[164]
	Hyaluronic acid, chitin, and chitosan	Glutaraldehyde	Catalase	*Bacillus subtilis*	25 cycles (73.80%) (79.55%)	[122]
	Fe_3_O_4_@chitosan	Glutaraldehyde	Laccase	*Rhus verniciflua*	10 cycles (75.8%)	[170]
	ZnO/chitosan	Glutaraldehyde, APTES	Trypsin	-	10 cycles (50%)	[171]
	Silica-coated magnetic nanoparticles	Isocyanatopropyltriethoxysilane	Prolidase	*Escherichia coli prolidase*	20 cycles (80%)	[172]
	Chitosan-Fe_3_O_4_ and chitosan-ZnO	-	α-Amylase	-	10 cycles (50%)	[173]
	Chitosan-cobalt oxide beads	Cyanuric chloride	Peroxidase	*Euphorbia tirucalli*	10 cycles (60%)	[174]
	Chitosan coated superparamagnetic nanoparticles	1,3,5-triazine	Glucoamylase	*Aspergillus niger*	10 cycles (70%)	[175]

APTES: (3-aminopropyl)triethoxysilane.

## 3. Enzyme Immobilization on Nanomaterials for Biotransformation

Immobilizing enzymes on nanomaterials involves anchoring enzyme molecules to solid support, increasing enzyme stability and reusability as well as facilitating product separation and purification. This process reduces the overall enzyme consumption and costs, which is economically feasible for industrial applications [176,177]. The interactions between enzymes and nanomaterial supports include physical interactions (van der Waals forces, hydrogen bonds, and hydrophobic interactions), covalent bonding, and affinity-based binding [28]. Immobilization techniques have received considerable attention because of their potential to increase enzyme performance and longevity in various industrial and biomedical applications [21]. The support matrix must be carefully selected for immobilizing enzymes, with options including inorganic, organic, and hybrid categories. Inorganic supports include silica, MNPs, and carbon-based materials, whereas organic supports include natural and synthetic polysaccharides [160,178,179]. Hybrid materials combine the advantages of both and offer enhanced properties compared with those of inorganic or organic materials alone. Nanomaterials provide significant advantages as enzyme supports over traditional organic and inorganic materials. Their exceptionally high surface area-to-volume ratio enables greater enzyme loading and stability, resulting in enhanced catalytic performance and reusability. Compared to current organic and inorganic supports, nanomaterials offer improved activity retention, broader operational conditions, and reduced mass transfer limitations, making them highly effective platforms for enzyme immobilization [80,116,117]. Research trends have shifted from traditional supports (alginate, chitosan, carrageenan, and starch) to newer materials such as CNTs and their derivatives, silica, magnetic particles, polyethylene glycol, and chitosan for immobilizing enzymes [180,181,182]. Various polymeric supports, magnetic composites, and nanomaterial-based carriers have been developed in the decade from 2015 to 2025. Nanomaterial-based enzyme immobilization is a powerful technique that increases enzyme stability, reusability, and catalytic efficiency in biotransformation processes.

Various nanomaterials, including CNTs, MNPs, and MOFs, have been used for immobilizing enzymes to increase performance and reusability. swCNTs and mwCNTs have shown considerable strengths in immobilizing enzymes owing to their large surface area, mechanical properties, and biocompatibility [183,184,185,186,187,188]. For example, Kumar et al. (2019) [63] immobilized lipase from mesoporous SiO_2_ microparticles, which were synthesized through spray pyrolysis using mwCNTs as a template, resulting in a template that could be used 12 times. Dense- and mesoporous-SiO_2_-bound lipases retained 74.2% and 95.4% of their initial activity, respectively [63]. MNPs, particularly Fe_3_O_4_, have received substantial attention owing to their superparamagnetic properties, which allow for easily separating and reusing immobilized enzymes [3]. Superparamagnetic properties are particularly advantageous for industrial applications because they enable efficiently recovering and recycling biocatalysts. For example, lipases immobilized on magnetic mwCNTs have increased activity and reusability in biotransformation processes in comparison of free enzyme. Lipase immobilized on CNTs filled with magnetic iron oxide and modified with polyamidoamine dendrimers had 17-fold higher specific activity than the free enzyme and retained 90% of its original activity after 20 cycles [183]. The lipase from *Burkholderia* sp. C20 was immobilized on alkyl-functionalized Fe_3_O_4_SiO_2_ magnetic nanoparticles for synthesizing biodiesel. The binding efficiency of the immobilized enzyme was 97%, and the maximum adsorption capacity was 29.45 mg g^−1^. The immobilized enzyme exhibited Michaelis–Menten kinetics for olive oil hydrolysis, with a *V_max_* of 6251 U g^−1^ and a *K_m_* of 3.65 mM. The immobilized enzyme converted >90% of the FAME from olive oil within 30 h using 11 wt% immobilized lipase for producing biodiesel. The biocatalyst maintained 90% of its activity after 10 use cycles, demonstrating high stability and reusability in transesterification reactions [189]. Polyamidoamine dendrimers were grafted onto magnetic mwCNTs to create a functionalized surface for the oriented immobilization of *Rhizomucor miehei* lipase (RML). The immobilized enzyme exhibited recovery activity up to 2808% and 27-fold higher esterification activity than the free enzyme.

Immobilized RML achieved 94% conversion of waste vegetable oil to biodiesel under optimized conditions. The immobilized RML enzyme maintained its catalytic efficiency over 10 cycles, with high stability and reusability. This immobilized RML system can be used as a robust and reusable catalyst for producing industrial biodiesel [69]. A packed-bed reactor was developed using a lipase-Fe_3_O_4_ nanoparticle biocomposite catalyst for producing biodiesel from soybean oil. Emulsification increased the reaction rate. Conversion remained at 45% after 240 h in the single-packed-bed reactor. A four-packed-bed system maintained >88% conversion for 192 h, dropping to 75% after 240 h, showing suitability for producing industrial-scale enzymatic biodiesel [190]. A lipase immobilization strategy using 5-aminoisophthalic acid grafted onto magnetic nanoparticles via Co^2+^ chelation. *Pseudomonas fluorescens* lipase had a 136.9 mg/g immobilization capacity and 2125% activity recovery, converting 95% of the waste cooking oil to biodiesel retaining 83% yield after 10 cycles. This support was easily regenerated and is therefore suitable for biotechnological applications [191]. The microfluidic bioreactor prototype enables small-scale enzymatic reactions in compartmented environments. Reactants immobilized on magnetic microcarriers facilitate separation and multiple reactions; permanent and alternating magnetic fields facilitate the separation and mixing. Kinetic studies using immobilized HRP revealed an enzyme activity of 89 U/g. Recycling experiments demonstrated the importance of magnetic resus pension, with yields of 65–95% over 10 cycles with resuspension compared with <10% without resuspension. The biochemical reactions in this system are efficient, controllable, and small in scale, so may be used in various applications [192]. Amano lipase from *P. fluorescens* was covalently immobilized on carbon nanomaterials (functionalized swCNTs and GO) for producing biodiesel. The most effective preparation using SWCNTNH_2_ derivatized with glycerol diglycidyl ether converted >99% of the sunflower oil in 4 h under optimal conditions. The biocatalyst retained over 99% of the initial activity after 20 reuse cycle in batch systems [193]. Salem et al. (2021) [123] immobilized microbial α-amylase (AmyKS) and xylanase (XAn11) on biomimetic MNPs (BMNPs) and inorganic MNPs using electrostatic interaction and covalent bonding. AmyKS was successfully immobilized on the BMNPs via electrostatic interactions, whereas XAn11 was immobilized on the MNPs via EDC/NHS crosslinking. A total of 92% of AmyKS and 87% of XAn11 were immobilized on BMNPs and MNPs–E/N under optimized conditions, respectively. AmyKS-BMNPs and XAn11-MNPs-E/N were highly reusable, retaining 82% and 64% of their initial activity after 15 and 11 reaction cycles, respectively [30]. MOFs have also been explored for immobilizing enzymes, with zeolitic imidazolate frameworks (ZIFs), showing promise for immobilizing enzymes on CNT surfaces [189]. The immobilization strategies include covalent binding, physical adsorption, and encapsulation, each offering unique advantages depending on the specific enzyme and its application. Nanomaterial-based immobilization techniques have substantially increased enzyme stability, activity, and reusability across various applications [194,195]. These examples demonstrate how enzyme immobilization on nanomaterials directly enhances biotransformation processes by improving the catalytic efficiency, stability, and reusability of biocatalysts [190,191]. For example, lipases immobilized on mesoporous SiO_2_ and magnetic nanoparticles (MNPs) enable efficient transesterification reactions for biodiesel production, achieving high conversion rates and sustained activity over multiple cycles [63]. Functionalized carbon nanotubes (CNTs) and MNPs not only increase enzyme loading and specific activity but also facilitate product separation and enzyme recovery, which are crucial for continuous biotransformation in industrial settings [183]. Microfluidic bioreactors with immobilized enzymes exemplify scalable, compartmentalized biotransformation, allowing precise control over reaction conditions and repeated substrate conversion [192]. These advancements in nanomaterial-based enzyme immobilization have implications for many industries, such as biofuel production, food processing, and pharmaceuticals, where efficient and reusable biocatalysts are highly desirable [3,196]. Further improvements in enzyme immobilization techniques are expected as research in this field continues, leading to more sustainable and cost-effective biotransformation processes.

## 4. Perspectives

Future research on nanomaterials will focus on three major areas: developing multifunctional nanomaterials, optimizing the immobilization methods, as well as biosafety and environmental concerns. First, the future of immobilizing enzymes on nanomaterials involves developing multifunctional supports that enhance enzyme stability, activity, and reusability. Researchers have focused on creating nanomaterials with properties tailored for specific enzymes and applications [3,17,23,28]. One future research direction involves synthesizing MNPs that are more biocompatible with functionalizable surfaces. These advanced MNPs will have higher enzyme-loading capacity as well as facilitate separation and reuse in industrial processes. Another future area of research includes focusing on developing hybrid nanomaterials that combine the advantages of different materials. For example, carbon-based nanomaterials coated with metal nanoparticles can have a large surface area and act as highly stable [30,63,169,197], producing synergistic effects and enhancing the overall performance of the immobilized enzymes. Second, future research should focus on refining immobilization techniques to maximize enzyme activity and stability. This could involve exploring new methods of attaching enzymes to supports, such as affinity-based binding and site-specific immobilization [160,198]. These approaches can help preserve the native structure and function of enzymes, thus increasing catalytic efficiency. Researchers are also developing more standardized and scalable immobilization protocols by optimizing parameters such as pH, temperature, and reaction time for different enzyme–nanomaterial combinations [80,196]. Computational modeling and high-throughput screening techniques could accelerate this optimization process, allowing for rapidly identifying the ideal immobilization conditions. Another avenue involves developing stimuli-responsive nanomaterials that modulate enzyme activity in response to external triggers [3,117]. This could increase the precision of the control of enzymatic reactions in industrial applications. Third, addressing safety and environmental concerns will be crucial as the industrial adoption of nanomaterial-immobilized enzymes becomes widespread. Future research should focus on developing green methods for synthesizing nanomaterials to minimize their environmental impact [119,199]. These may include the use of plant extracts, microorganisms, and other eco-friendly approaches for producing nanomaterials with reduced toxicity. Long-term studies must be conducted on the release of nanoparticles from immobilized enzyme systems and their environmental fate [157,200]. These studies will help with establishing guidelines for the safe use and disposal of nanobiocatalysts in industrial settings. Additionally, developing biodegradable nanomaterials as enzyme supports could address concerns regarding their environmental accumulation. These materials could naturally break down after their useful lives, reducing their long-term environmental impacts. VOSviewer constructs and visualizes bibliometric networks for analysis; shows research trends, hot topics, and keyword relationships; and helps to identify active areas, emerging directions, and core themes within a scientific research field. It also shows if the number of scientific studies on nanomaterial synthesis and their application in various sectors has increased annually, which aids researchers in understanding research advancements in the field. Another target for future research will likely be engineering nanomaterials that can mimic or enhance natural enzyme environments, improving selectivity and conversion rates for complex biotransformations. The integration of nanomaterials with synthetic biology enables the design of tailored biocatalysts for specific chemical conversions, including those relevant to pharmaceuticals, fine chemicals, and environmental remediation. Moreover, advances in in situ monitoring and real-time control of biotransformation reactions using nanomaterial-based sensors will facilitate process optimization. These developments are expected to expand the scope and efficiency of biotransformation applications, making them more sustainable and economically viable [157,160,198,199,200].

## 5. Conclusions

Nanomaterial-immobilized enzymes are promising for industrial and biomedical applications. Researchers can ensure the full potential of nanobiocatalysts can be exploited by developing multifunctional nanomaterials, optimizing immobilization protocols, and addressing safety and environmental concerns. These nanomaterial-immobilized enzymes could increase the efficiency and sustainability of biofuel production, food processing, pharmaceutical production, and environmental remediation processes. Collaboration between materials scientists, enzymologists, and environmental researchers will be crucial as the field advances to overcome the current limitations and realize the full potential of nanomaterial-immobilized enzymes. These innovative biocatalysts are expected to play a large role in shaping the future of sustainable industrial processes and biotechnological applications with continued research and development.

## Figures and Tables

**Table 1:** Advantages and disadvantages of nanomaterials used for immobilizing enzymes.

Advantage	Disadvantage	Solution
**High surface area and loading capacity** Nanomaterials, such as CNTs, MNPs, and graphene, have a large surface area-to-volume ratio, increasing enzyme loading and catalytic efficiency [3,5,6].	**High cost of nanomaterials** Fabrication and functionalization of nanomaterials are expensive; large-scale applications are economically challenging [5,7,8].	Adopt low-cost fabrication techniques such as plasma reactors and sol–gel methods.
**Enhanced stability** Immobilizing nanomaterials increases enzyme stability against denaturation caused by environmental factors such as temperature, pH, and solvents, which are suitable for industrial applications [3,6,9].	**Diffusion limitations** Diffusion of substrates and products can be restricted within the immobilization matrix at high enzyme loading densities, reducing apparent activity [5,7,9].	Use of porous supports or optimize matrix design to increase diffusion.
**Reusability and cost-effectiveness** Enzymes immobilized on nanomaterials can be easily separated from reaction mixtures (e.g., MNPs can be recovered using external magnetic fields) and reused for multiple cycles, reducing operational costs [3,6,10].	**Enzyme leaching and instability** Noncovalent immobilization methods may result in enzyme leaching over time, reducing catalytic performance and contaminating the reaction medium [7,9].	Use covalent binding or crosslinking to enhance enzyme immobilization stability.
**Increase catalytic activity** Nanoscale interactions between enzymes and nanomaterials enhance catalytic activity due to orientation and stabilization of the enzyme’s active site [3,5].	**Complex preparation** Immobilization procedures often require specialized techniques and equipment, increasing process complexity compared with traditional methods [7,8].	Develop simplified protocols or use prefunctionalized, ready-to-use supports.
**Versatility in applications** Enzymes immobilized on nanomaterials are used in various fields such as biosensors, biofuel production, drug delivery, and industrial biocatalysis due to their functionality [3,6].	**Scalability issues and environmental sensitivity** Scalability of enzyme immobilization on nanomaterials is limited due to challenges with reproducibility and uniformity during large-scale production [8,11]. Some nanomaterials are prone to degrading or aggregating under specific conditions (e.g., MNPs in acidic or oxidative environments), which can compromise enzyme stability [3,7].	Optimization of processes and development of novel stable, environmentally resistant nanomaterials for immobilization.

**Table 2:** Methods of enzyme immobilization as well as their advantages and disadvantages.

Method	Advantages	Disadvantages	References
**Adsorption**	Simple and cost-effective method. Minimal chemical modification of enzyme. Rapid immobilization process.	Adsorption immobilization relies on weak binding forces (van der Waals, ionic, and hydrogen bonds), making enzymes prone to desorption under shifts in pH, ionic strength, or temperature, resulting in low stability and activity loss.	[20,21]
**Covalent Binding**	Strong and stable linkage prevents enzyme leakage. High thermal and operational stability. Reusable for multiple cycles.	Covalent binding of enzymes presents significant drawbacks, primarily the risk of enzyme denaturation due to chemical modifications during immobilization. This method involves a complex process requiring expensive reagents and supports, increasing operational costs. Additionally, conformational changes induced by binding often lead to partial or complete loss of enzyme activity, reducing catalytic efficiency.	[8,14,22,23,25].
**Entrapment**	Protects enzymes from environmental changes (e.g., pH and temperature). Suitable for thermally and mechanically stable enzymes.	Diffusion limitations restrict substrate access to enzymes, reducing reaction rates. Recovering enzymes is challenging as they are trapped within the matrix, complicating reuse and process efficiency.	[14]
**Encapsulation**	High reproducibility and protection from shear forces.	Encapsulation includes weak binding forces that can cause enzyme leakage, particularly under certain conditions such as high ionic strength. Enzyme inactivation due to mechanical stress during the encapsulation process is also a risk. Additionally, swelling of the capsules can lead to further leaching of the encapsulated enzymes. Low mechanical strength and large pore size in some encapsulation materials can also contribute to high enzyme leakage.	[15,26]
**Crosslinking**	Carrier-free immobilization with high enzyme density. High stability under industrial conditions. Easy recycling and reuse.	Disadvantages of crosslinking include possible overcrowding of enzymes, which can reduce their activity. The process is irreversible, meaning that once enzyme activity is lost, it cannot be recovered. Additionally, crosslinking often requires highly pure, crystallized enzymes, making it an expensive technique. The chemicals used can also result in enzyme denaturation, further inhibiting activity.	[23,27]

**Table 3:** CNTs and their derivatives used for immobilizing enzymes.

Nanomaterial	Skeleton Matrix	Functionalization Groups	Enzyme	Enzyme Source	Reusability (Retained Activity)	Reference
**CNT and derivatives**	Multiwalled CNT (mwCNT)	HNO_3_/H_2_SO_4_	Laccase	*Trametes versicolor*	10 cycles (90%)	[49]
	mwCNT	EDC/NHS crosslinkers	Lipase	*Pseudomonas fluorescens*	10 cycles (49.2%)	[50]
	CNT	N-aminoethyl-*γ*-aminopropyl trimethoxy	*β*-glucosidase	*Terrabacter ginsenosidimutans*	14 cycles (76%)	[51]
	mwCNT-NiO	Glutaraldehyde, APTES	Xylanase	*Thermomyces lanuginosus*	6 cycles (80%)	[52]
	mwCNT	HNO_3_/H_2_SO_4_	Laccase	*Trametes versicolor*	10 cycles (90%)	[49]
	Fe_3_O_4_ mwCNT	EDC/NHS	Lipase	*Candida rugosa*	10 cycles (91%)	[53]
	Magnetic nickel oxide mwCNT	3-aminopropyltriethoxysilane	L-asparaginase	*Geobacillus kaustophilus*	10 cycles (90%)	[54]
	ZIF-8@MWCNT	HNO_3_/H_2_SO_4_	Laccases	*-*	10 cycles (68%)	[55]
	GO-CNT	Glutaraldehyde	Laccase	*Trametes versicolor*	11 cycles (50%)	[56]
	Superparamagnetic mwCNT	Polyethylene glycol amine polymer	Lipases	*-*	10 cycles (78.5%)	[48]
	CNT	Glutaraldehyde/APTES	Lipase	*Rhizomucor miehei*	10 cycles (90%)	[57]
	CNT	Cinnamaldehyde ethanol solution	Porcine pancreatic lipase	*Porcine pancreatic lipase*	7 cycles (69%)	[58]
	Polyaniline cobalt CNT	Glutaraldehyde	β-galactosidase	*Aspergillus oryzae*	10 cycles (74%)	[59]
	CNT	Glutaraldehyde	Laccase	*Novo Nordisk Company, Bagsværd, Denmark*	10 cycles (69%)	[60]
	mwCNT-MoS2 NC	Glutaraldehyde crosslinker	β-galactosidase	*Lens culinaris*	21 cycles (>50%)	[61]
	mwCNT	Glutaraldehyde	Cyanate hydratase	*Pichia pastoris*	10 cycles (>94%)	[62]
	Mesoporous SiO_2_ microparticle	Glutaraldehyde	Lipase	*Thermomyces lanuginosus*	12 cycles (95.4%)	[63]
	mwCNT	Aminated polydopamine	Lipase	*Candida rugosa*	10 cycles (84%)	[64]
	mwCNT	Sodium alginate	Cellulase	*Trichoderma*	7 cycles (70%)	[65]
	mwCNT	-	Lipase	*Candida antarctica*	7 cycles (95%)	[66]
	mwCNT	Carbodiimide coupling	Cellulase	*Aspergillus niger*	10 cycles (85%)	[67]
	swCNT	*N,N′*-carbonyldiimidazole, CH_2_Cl_2_	Lipase B	*Candida antarctica*	10 cycles (>90%)	[68]
	Magnetic-mwCNT	Polyamidoamine	Lipase	*Rhizomucor miehei*	10 cycles (94%)	[69]

EDC: 1-ethyl-3-(3-dimethylaminopropyl) carbodiimide, APTES: (3-aminopropyl)triethoxysilane, NHS: N-hydroxysulfosuccinimide.

**Table 4:** Graphene and derivatives used for immobilizing enzymes.

Nanomaterial	Skeleton Matrix	Functionalization Groups	Enzyme	Enzyme Source	Reusability (Retained Activity)	Reference
**Graphene and derivatives**	Graphene oxide (GO) Fe_3_O_4_@SiO_2_	Glutaraldehyde	Phospholipase D	*Streptomyces chromofuscus*	10 cycles (78.3%)	[81]
	Graphene oxide (GO-Asp-Fe_3_O_4_)	EDC/NHS	L-asparaginase	*-*	8 cycles (80%)	[82]
	Magnetic GO	3-Mercaptopropyl trimethoxysilane	Lipase	*Candida rugosa*	8 cycles 76.5%	[83]
	Magnetic GO	Glutaraldehyde	Lipase	*Candida rugosa*	10 cycles (50%)	[84]
	Magnetic GO	γ-Ureapropyltrimethoxy silane, APTES, γ-mercapto propyltriethoxysilane	Lipase	*Aspergillus oryzae*	8 cycles (80.2%)	[85]
	Activated GO/chitosan/cellulose	Glutaraldehyde	Lipase	*Bacillus licheniformis (Lipase Km12)*	10 cycles (80%)	[86]
	Magnetic GO	Carbodiimide	Cellulase/sylanase	*-*	10 cycles (70%)	[87]
	Magnetic GO	Glutaraldehyde, APTES	PersiManXyn1	*-*	15 cycles (94%)	[88]
	Magnetic GO	Polyethylenimine	Lactase	*Escherichia coli*, Sangon Biotech (Shanghai, China)	20 cycles (83.1%)	[89]
	Magnetic GO	Polyethylene glycol (PEG)	Horseradish peroxidase	*-*	8 cycles (68.1%)	[90]
	Magnetic GO	Glutaraldehyde	Dextranase	*-*	20 cycles (85.7%)	[91]
	GO	Glutaraldehyde	Horseradish Peroxidase	*-*	12 cycles (90%)	[70]
	GO	Horseradish peroxidase and oxalate oxidase	Formate dehydrogenase	*Candida boidinii*	8 cycles (63.8%)	[92]
	Reduced GO	Glutaraldehyde	Horseradish peroxidase	*-*	10 cycles (70%)	[93]
	Magnetic GO	Nα, Nα-Bis(carboxymethyl)-l-lysine hydrate	Laccase	*-*	10 cycles (89.4%)	[94]
	GO	Amine group	Horseradish peroxidase	*-*	10 cycles (60%)	[95]
	GO	-	Lipase	*Candida rugosa*	10 cycles (85%)	[96]
	Reduced GO	-	Laccase, horseradish peroxidase	*Trametes versicolor*	10 cycles (92.6%)	[97]
	Graphene	-	Naringinase		10 cycles (85.4%)	[98]
	Polyaniline–silver functionalized GO nanocomposites	Glutaraldehyde	Lipase	*Aspergillus niger*	11 cycles (86%)	[99]
	Magnetic GOx	EDC/NHS	Laccase	*Trametes versicolor*	11 cycles (59.8%)	[100]
	GO(GO/ZnO)	-	Lipase	*Candida rugosa*	14 cycles (90%)	[101]
	GO	EDAC, *N*-hydroxysulfosuccinimide sodium salt	Chloroperoxidase	*Caldariomyces fumago*	8 cycles (52%)	[102]
	GO	Epoxy chloropropane	γ-Lactamase	*Escherichia coli BL21(DE3)*	15 cycles (70%)	[103]
	GO nanosheets Superparamagnetic iron oxide nanoparticles	Cyanuric chloride	Xylanase	*Thermomyces lanuginosus*	10 cycles (70%)	[104]
	Graphene–iron oxide nanocomposites (Gr@Fe_3_O_4_ NCs)	-	β-galactosidase	*Aspergillus oryzae*	8 cycles (83%)	[105]
	Graphene sheets	Cysteamine and glutaraldehyde	α-Amylase	*Triticum aestivum*	10 cycles (73%)	[106]
	Exfoliated graphene oxide (EGO)	Silane	Lipase	*Candida rugosa*	30 cycles (50%)	[107]
	GO-based magnetic	3-Chloropropyltriethoxysilane	Lipase	*-*	10 cycles (87%)	[108]

EDC: 1-ethyl-3-(3-dimethylaminopropyl) carbodiimide, APTES: (3-aminopropyl)triethoxysilane, NHS: N-hydroxysulfosuccinimide.

**Table 5:** Metal and metal oxide nanoparticles used for immobilizing enzymes.

Nanomaterial	Skeleton Matrix	Functionalization Groups	Enzyme	Enzyme Source	Reusability (Retained Activity)	Reference
**Metal and metal oxide nanoparticles**	Magnetic laccase nanoflowers	Glutaraldehyde	Laccase	*Trametes versicolor*	18 times (90%)	[121]
	Metal–organic frameworks	Glutaraldehyde	α-Amylase	*Bacillus subtilis*	20 cycles (81%)	[122]
	Magnetic nanoparticles	Glutaraldehyde	α-Amylase and xylanase	*Bacillus subtilis and Aspergillus niger*	15 cycles (82%) 11 cycles (64%)	[123]
	Fe_3_O_4_/SBA-15	Tannic acid	L-asparaginase	*Escherichia coli*	16 times (70%)	[124]
	Titania nanoparticles	Glutaraldehyde	Alcohol dehydrogenase	*Saccharomyces cerevisiae*	10 times (84%)	[125]
	-	Naringin	α-Amylase	*Bacillus subtilis*	10 times (60%)	[126]
	FeCl_3_	APTES, Glutaraldehyde	Laccase	*Aspergillus oryzae*	6 cycles (75%)	[127]
	Magnetic nanoparticles	3-phosphono propionic acid	α-Amylase	*Aspergillus oryzae*	10 cycles (60%)	[128]
	Fe_3_O_4_ magnetic nanoparticles	Polyethylenimine	Lipase	*Thermomyces lanuginosa*	10 cycles (60%)	[129]

APTES: (3-aminopropyl)triethoxysilane.

**Table 6:** Magnetic nanoparticles (MNPs) used for immobilizing enzymes.

Nanomaterial	Skeleton Matrix	Functionalization Groups	Enzyme	Enzyme Source	Reusability (Retained Activity)	Reference
**Magnetic nanoparticles (MNPs)**	Silica-coated amine functionalized iron oxide nanoparticle (IONP@SiO_2_-NH_2_)	Glutaraldehyde	Cellulase	*-*	6 cycles (80%)	[134]
	Fe_3_O_4,_ Affi-Cova beads	IDA	r-BirA		10 cycles (76.1%)	[37]
	Fe_3_O_4,_ SiO_2_ core@shell	3-(Triethoxysilyl) propyl isocyanate	Laccase	*Trametes versicolor*	13 cycles (88%)	[131]
	-	Glutaraldehyde	Trypsin	*-*	15 cycles (93%)	[135]
	Fe_3_O_4_	N-hydroxysuccinimide, ethyl-3-(3-dimethylaminopropyl) carbodiimide	β-Lactamase	*Bacillus cereus*	12 cycles (BC: 57%, IC: 65%)	[136]
	Fe_3_O_4_	N-acetyl and N-methylamide	Lipase	*Thermomyces lanuginosus*	12 cycles (80%)	[137]
	Fe_3_O_4_	SpyCatcher-fused elastin-like polypeptides	Xylanase Lichenase	*-*	10 cycles (66.3 and 72.7%)	[138]
	Fe_3_O_4,_ SiO_2_-CHO	Tetraethyl orthosilicate, APTES, and glutaraldehyde	Lipase	*Saitozyma podzolica*	10 cycles (90%)	[139]
	Fe_2_O_3,_ Fe_3_O_4_	APTES	Laccase		10 cycles (82.9%)	[140]
	Fe_3_O_4_	Hyaluronic acid	Isocitrate dehydrogenases	*-*	DH/HA/MNPs, IDH/HA/MNPs-CLEAs and IDH/BSA/HA/MNPs-CLEAs 15 cycles (76%, 80%, 85%)	[141]
	Super paramagnetic iron oxide nanoparticles	APTES	Laccase	*Thermomyces lanuginosus*	12 cycles (80%)	[137]
	Fe_3_O_4_@C@cellulase-SiO_2_	-	Cellulase	*-*	9 cycles (80%)	[142]
	Fe_3_O_4_	Hydroxyapatite in cobalt ferrite (CoFe_2_O_4_)	β-glucosides	*-*	10 cycle (70%)	[143]
	Fe_3_O_4,_ carboxymethyl cellulose	PPL-MCMC PPL-IL-MCMC	Cellulose	*-*	10 cycles (83.9%, 86.1%)	[144]
	Fe_3_O_4,_ chitosan	Glutaraldehyde	Peroxidase	*Pseudomonas aeruginosa*	100 cycles (95%)	[145]
	His_6_-*Ec*PepQ@NiNTASiMNPs	Ni^2+^	Lipase	*Thermomyces lanuginosus*	20 cycles (80%)	[146]
	FeCl_2,_ FeCl_3_	Glutaraldehyde, glycidol	Chitosan	*Aspergillus niger*	15 cycles (80%)	[147]

APTES: (3-aminopropyl)triethoxysilane.

## Data Availability

The data supporting this article have been collected from the following sources: Data for Figure 3 were obtained from the Pubmed database (https://pubmed.ncbi.nlm.nih.gov/), accessed in 10 February 2025, and were analyzed and visualized using VOSviewer (https://app.vosviewer.com/) accessed in 10 February 2025. All data used is publicly available from these databases.
